# Salvianolic Acid B Improves Postresuscitation Myocardial and Cerebral Outcomes in a Murine Model of Cardiac Arrest: Involvement of Nrf2 Signaling Pathway

**DOI:** 10.1155/2020/1605456

**Published:** 2020-07-01

**Authors:** Qing-Qi Ji, Yan-Jie Li, Ying-Hua Wang, Zi Wang, Liang Fang, Lan Shen, Yan-Qiao Lu, Ling-Hong Shen, Ben He

**Affiliations:** ^1^Department of Cardiology, Shanghai Chest Hospital, Shanghai Jiao Tong University, Shanghai 200030, China; ^2^Department of Intensive Care Unit, Shanghai Chest Hospital, Shanghai Jiao Tong University, Shanghai 200030, China; ^3^Department of Cardiac Surgery, Shanghai Chest Hospital, Shanghai Jiao Tong University, Shanghai 200030, China

## Abstract

Survival and outcome of cardiac arrest (CA) are dismal despite improvements in cardiopulmonary resuscitation (CPR). Salvianolic acid B (Sal B), extracted from Salvia miltiorrhiza, has been investigated for its cardioprotective properties in cardiac remodeling and ischemic heart disease, but less is known about its role in CA. The aim of this study was to learn whether Sal B improves cardiac and neurologic outcomes after CA/CPR in mice. Female C57BL/6 mice were subjected to eight minutes of CA induced by an intravenous injection of potassium chloride (KCl), followed by CPR. After 30 seconds of CPR, mice were blindly randomized to receive either Sal B (20 mg/kg) or vehicle (normal saline) intravenously. Hemodynamic variables and indices of left ventricular function were determined before CA and within three hours after CPR, the early postresuscitation period. Sal B administration resulted in a remarkable decrease in the time required for the return of spontaneous circulation (ROSC) in animals that successfully resuscitated compared to the vehicle-treated mice. Myocardial performance, including cardiac output and left ventricular systolic (dp/dt_max_) and diastolic (dp/dt_min_) function, was clearly ameliorated within three hours of ROSC in the Sal B-treated mice. Moreover, Sal B inhibited CA/CPR-induced cardiomyocyte apoptosis and preserved mitochondrial morphology and function. Mechanistically, Sal B dramatically promoted Nrf2 nuclear translocation through the downregulation of Keap1, which resulted in the expression of antioxidant enzymes, including HO-1 and NQO1, thereby counteracted the oxidative damage in response to CA/CPR. The aforementioned antiapoptotic and antioxidant effects of Sal B were impaired in the setting of gene silencing of Nrf2 with siRNA in vitro model. These improvements were associated with better neurological function and increased survival rate (75% vs. 40%, *p* < 0.05) up to 72 hours postresuscitation. Our findings suggest that the administration of Sal B improved cardiac function and neurological outcomes in a murine model of CA via activating the Nrf2 antioxidant signaling pathway, which may represent a novel therapeutic strategy for the treatment of CA.

## 1. Introduction

Sudden cardiac arrest (CA) is one of the leading causes of death in adults worldwide, despite significant improvements in cardiopulmonary resuscitation (CPR) techniques over recent years. Based on the American Heart Association's “Heart Disease and Stroke Statistics—2019 Update,” there are approximately 356,000 cases of out-of-hospital CA and 209,000 cases of in-hospital CA in the United States each year [1]. In China, the situation is even worse, and mortality is considerable. Therefore, active protection of both cardiac and neurologic function is critical for the improvement of postresuscitation outcomes.

Danshen, the dried root of Salvia miltiorrhiza, is considered one of the most important traditional Chinese medicines. Traditionally, Danshen is used to improve body function via the promotion of microcirculation, as well as to treat insomnia, dysmenorrhea, hemorrhage, hepatitis, and miscarriage [2–6]. More recently, a body of clinical trials investigated its protective effect on cardiovascular risk factors in patients with hypertension, hyperlipidemia, and diabetes [7–9]. Salvianolic acid B (Sal B), the major water-soluble ingredient in Danshen, has been extensively used in eastern countries such as China and, to a lesser extent, the United States and Europe for the prevention and treatment of cardiovascular and cerebrovascular diseases. A variety of studies have reported that the main biological activities of the whole-plant Danshen herb are attributed to Sal B, the most bioactive constituent of Danshen [10, 11]. Sal B treatment can attenuate ischemia/reperfusion (I/R) injury in the heart [12], brain [13], liver [14], and kidney [15]. Modern pharmacology studies have revealed that Sal B exerts diverse pharmacological effects on the cardiovascular system. The protective effects of Sal B appear to be mediated via antiapoptotic, antioxidative, and/or anti-inflammatory effects. However, the impact of Sal B on the outcomes of postresuscitation, which is complicated by whole-body I/R injury, has hitherto remained obscure.

Nuclear factor (erythroid-derived 2)-related factor 2 (Nrf2) is a major transcription factor that is integral in inducing antioxidant enzymes to combat oxidative stress [16]. Under normal conditions, Nrf2 is targeted by Kelch-like ECH-associated protein 1 (Keap1) in the cytoplasm that promotes Nrf2 degradation via interactions with a ubiquitin ligase [17]. When under oxidative stress, Nrf2 undergoes modifications which compromise Keap1/Nrf2 interactions, leading to the dissociation of Nrf2 from Keap1 complex, and then translocates to the nucleus. After entering the nucleus, Nrf2 binds to antioxidant response elements to trigger the transcription of antioxidant enzymes, including heme oxygenase-1 (HO-1) and NADPH: quinone oxidoreductase 1 (NQO1), exerting antiapoptosis, anti-inflammatory, and antitumor effects [18]. Accumulating evidence has implicated the importance of Nrf2 signaling pathway in the antioxidant defense system in cardiovascular diseases such as myocardial I/R injury, hypertension, and chronic inflammation [19]. Recent studies indicate that Sal B involved in the activation of the Nrf2 signaling pathway in multiple diseases [20–22]. However, little information is available regarding the relationship between Sal B and Nrf2 signaling pathway in ischemic heart disease. Hence, we investigated whether Sal B has cardioprotective effects on post-CA myocardial dysfunction and tried to elucidate the potential mechanisms involved.

In this novel study, we demonstrated that the administration of Sal B during the early CPR period attenuated myocardial injury and apoptosis, which prevented post-CA myocardial dysfunction, reduced end-organ damage, and improved neurological function and survival in mice. The salutary impact of Sal B on the outcomes of postresuscitation was associated with the inhibition of oxidative stress via activating the Nrf2 pathway in the heart.

## 2. Materials and Methods

### 2.1. Reagents and Antibodies

Sal B was purchased from MedChemExpress (Monmouth Junction, NJ, USA). Potassium chloride (KCl) was obtained from Sigma (St. Louis, MO, USA). Dihydroethidium (DHE) was from Life Technologies (Carlsbad, CA, USA). Protease inhibitor cocktail was from Sigma Chemicals (St. Louis, MO, USA). Antibodies against gp91^phox^ (1 : 1000, sc-130543), Keap1 (1 : 500, sc-515432), and NQO1 (1 : 500, sc-32793) were obtained from Santa Cruz Biotechnology (Santa Cruz, CA, USA); Cleaved caspases-3 (1 : 500, ab49822) and Nrf2 (1 : 1000, ab92946) were from Abcam (Cambridge, UK); HO-1(1 : 1000, AF1333) and Histone H3 (1 : 500, AF7101) were from Beyotime Institute of Biotechnology (Shanghai, China); GAPDH (1 : 2000; #2118), Bax (1 : 1000, #14796), and Bcl-2 (1 : 1000, #3498) were from Cell Signaling Technology (Beverly, MA, USA).

### 2.2. Animals and the Establishment of a Cardiac Arrest and Cardiopulmonary Resuscitation (CA/CPR) Model

Animal housing and treatments were performed in adherence to the guidelines for the Care and Use of Laboratory Animals published by the United States National Institutes of Health (8th Edition, 2011), and all protocols were approved by the Institute's Animal Ethics Committee of Shanghai Jiao Tong University. Wild-type (WT) C57BL/6 female mice (8-10 weeks old, 21-25 g) were purchased from the Slac Laboratory (Shanghai, China) and were fed a standard rodent diet with free access to food and tap water. The housing conditions were as follows: temperature of 24 ± 1.5°C, relative humidity of 50%, and a 12-hour light/dark cycle. After terminal studies at the indicated time points, animals were euthanized under isoflurane inhalation (2.0%) followed by cervical dislocation in compliance with the American Veterinary Medical Association Guidelines on Euthanasia.

The CA/CPR model was created as described previously [23, 24]. We utilized the murine model of KCl-induced CA because it afforded a reproducible method of precise timing of CA with little electrical injury compared to the induction of CA by asphyxia and electrical defibrillation [23]. Mice were anesthetized via inhalation of 2.0% isoflurane and maintained under anesthesia with 1.0% isoflurane/oxygen. Rectal temperature was continuously recorded and maintained at 37.0 ± 0.5°C during surgery with a heating pad. Mice were then intubated with an endotracheal catheter attached to a rodent ventilator (MiniVent 845, Harvard Apparatus, Holliston, MA, USA). For fluid administration, a 0.61 mm outer diameter PE tube (SDR Scientific, Sydney, Australia) was inserted into the right jugular vein and flushed with heparinized normal saline. For mice undergoing invasive hemodynamic monitoring, a conductance pressure-volume catheter (SPR-839, Millar Instruments, USA) was placed into the left ventricle via the carotid artery, and needle-probe electrocardiogram (ECG) monitoring was initiated throughout the experimental procedures. CA was induced by an injection of 0.08 mg KCl/g body weight through the jugular catheter, and the endotracheal catheter was disconnected from the ventilator. Following eight minutes of CA, the ventilator was reconnected, and chest compressions were delivered with a finger at a rate of 350 to 400 beats per minute (bpm). After 30 seconds of CPR, 0.4 *μ*g epinephrine/g body weight combined with 20 mg Sal B/kg body weight dissolved in normal saline, or epinephrine plus normal saline only, was injected through the venous line. This dose of Sal B was chosen based on our pilot study data and a previously published report [25]. The return of spontaneous circulation (ROSC) was defined as the return of sinus rhythm in conjunction with a mean arterial pressure above 40 mmHg that lasted at least five minutes. CPR was terminated when ROSC was achieved or after five minutes of unsuccessful CPR. Mice in which ROSC failed to occur within five minutes were excluded from the continuing studies. To prevent dehydration, resuscitated animals received a 0.5 mL normal saline subcutaneous injection. Mice were weaned from mechanical ventilation two hours after CPR and returned to the animal facility with free access to water and food.

### 2.3. Echocardiography

Cardiac function and structure were assessed three hours post-ROSC using echocardiography (Vevo 2100, VisualSonics, Toronto, Canada) with a 25 MHz imaging transducer. Mice were anesthetized by inhalation of 2% isoflurane and oxygen. Measurements of left parasternal long axes and M-mode images were acquired at a heart rate of 450-500 bpm. The left ventricular ejection fraction (LVEF) and left ventricular fractional shortening (LVFS) were calculated as previously described [26]. Body temperature was maintained with the aid of a heating pad.

### 2.4. Histology Staining

Three hours after ROSC, the heart was collected, and fixed in 4% paraformaldehyde overnight, dehydrated, and embedded in paraffin. Five-micron sections of the heart were prepared, deparaffinized and blocked, stained with hematoxylin and eosin, and then visualized by light microscopy.

### 2.5. Transmission Electron Microscopy

Morphological changes in the myocardial mitochondria were evaluated using transmission electron microscopy. Briefly, the left ventricle was isolated and sliced into thin sections, and the slices were fixed with 2.5% glutaraldehyde overnight. The samples were rinsed with phosphate-buffered saline (PBS), fixed in cacodylate-buffered osmium tetroxide (OsO_4_), dehydrated, and embedded in epoxy resin. Ultrathin sections (100 nm) were cut with an ultramicrotome (RMC/MTX, Elexience), mounted on mesh copper, contrasted with 10% uranyl acetate and lead citrate, and examined under an electron microscope (Philips CM-120, Philips Electronic Instruments, Netherland).

### 2.6. TUNEL Staining

TUNEL staining was performed using an In Situ Cell Death Detection Kit (Roche, Mannheim, Germany) according to the manufacturer's instructions. The nuclei were identified by DAPI (blue), and the apoptotic cells stained with green. The TUNEL-positive nuclei were calculated as the percentage (%) of total nuclei. Images were obtained using the Leica laser fluorescence microscope at a magnification of ×200 and analyzed with Image-pro plus 6.0.

### 2.7. Determination of Cardiac and Cellular Oxidative Stress Generation

For the detection of superoxide generation in the myocardium, DHE staining and lucigenin-enhanced chemiluminescence were used. Three hours after successful ROSC, the mice were sacrificed and the hearts were harvested and directly embedded in OCT. Fresh frozen heart sections (4-5 *μ*m) were incubated with 5 *μ*M DHE for 30 minutes at 37°C in a humidified chamber protected from the light. The reaction was terminated by three washes in 1X PBS, and images were acquired by a Leica fluorescent microscope. Nicotinamide adenine dinucleotide phosphate (NADPH) oxidase activity in cardiac tissues was assessed by a lucigenin-enhanced chemiluminescence assay according to the manufacturer's instructions. NADPH oxidase activity was calculated as the relative light unit changes per mg of heart tissue weight per second (RLU/mg/s). For the measurement of the production of cellular superoxide, H9c2 cardiomyocytes were incubated with 5 *μ*M DHE for 30 min at 37°C protected from light. After incubation, cells were observed under a Leica fluorescent microscope.

### 2.8. siRNA Transfection, Hypoxia/Reoxygenation (H/R) Cell Model, and Sal B Treatment

The H9c2 cardiomyocytes were obtained from the American Type Culture Collection (ATCC). Nrf2 was silenced in H9c2 cardiomyocytes by using Nrf2 siRNA. The rat siRNA targeting at Nrf2 and control siRNAs was purchased from Santa Cruz Biotechnology Inc. (California, USA) and transfected to the cells using Lipo3000 (Invitrogen, Massachusetts, USA). After 72 hours transfection, the following groups were investigated: (1) Blank group: the cells without any treatment were cultured in a standard incubator; (2) NC group: the cells were transfected with negative control siRNA and exposed to hypoxia (95% N2/5% CO2) for 6 hours and reoxygenation for 12 hours; (3) Nrf2 siRNA group: the cells were transfected with Nrf2 siRNA and exposed to hypoxia for 6 hours and reoxygenation for 12 hours; (4) NC + Sal B group: the cells were transfected with negative control siRNA and exposed to hypoxia for 6 hours, and then treated with Sal B (10 *μ*M) prior to and during reoxygenation for 12 hours; and (5) Nrf2 siRNA+Sal B group: the cells were transfected with Nrf2 siRNA and exposed to hypoxia for 6 hours, and then treated with Sal B (10 *μ*M) prior to and during reoxygenation for 12 hours.

### 2.9. Western Blot Analysis

Western blot analysis was carried out using lysates from hearts collected three hours following ROSC. Cytoplasmic and nuclear and fractions were extracted using NE-PER Nuclear and Cytoplasmic Extraction Reagents (Thermo Fisher Scientific). For the whole-cell protein extraction, the heart tissues were homogenized in radio-immunoprecipitation assay lysis buffer containing 150 mM NaCl, 10% Triton X-100, 0.5% Sodium deoxycholate, 0.1% SDS, 50 mM Tris base, and a protease inhibitor cocktail. Then, the homogenate was centrifuged at 14000 × g for 20 minutes at 4°C. The supernatant was collected, and the protein concentration was determined using a BCA protein concentration assay kit. Equal amounts (40 *μ*g) of denatured proteins from each sample were subjected to 10% SDS-PAGE and transferred to a nitrocellulose membrane (GE Healthcare Life Science, NJ, USA). The membranes were then blocked with 5% defatted milk for 1 hour at room temperature and probed with primary antibodies overnight at 4°C. After a 1-hour incubation with HRP-conjugated secondary antibodies, the labeled proteins were detected by enhanced chemiluminescence and quantified by their scanning intensity with the Gel-Pro Analyzer software version 4.0 (Media Cybernetics, Silver Spring, MD, USA).

### 2.10. Survival Analysis and Assessment of Neurological Function

To confirm the mortality outcome, which was the endpoint of the survival analysis for CA/CPR, mortality in the different groups was monitored for 72 hours. Mortality was confirmed by the cessation of spontaneous respiratory movements and heartbeat that persisted for at least two minutes. Neurological function in each group undergoing survival analysis was determined within 72 hours after ROSC with the use of a validated neurological function scoring system as reported previously [23]. Scoring was performed by two independent investigators who were blinded to the treatment group. In case of discrepancies, a third observer was invited to participate, and the score chosen was accepted by the majority. The scoring was evaluated using six criteria (response to pinching of tail, corneal reaction, respiration, righting reflex, coordination, and movement). Each criterion was rated on a 0 (absent function) to 2 (normal function) numerical rating scale. The highest possible score was 12, which indicated the normal behavior in the mice.

### 2.11. Statistical Analyses

Data are presented as means ± SD. Statistical analysis was conducted using SPSS software version 23.0 (SPSS, Chicago, IL, USA). Comparisons between the two groups or multiple groups were performed using unpaired *t*-test or one-way ANOVA with Bonferroni post-hoc tests, as appropriate. Categorical data were presented as percentages and analyzed using continuity corrected Pearson Chi-square tests. Time courses of variables were compared by repeated-measures two-way ANOVA followed by Bonferroni comparison post-hoc tests. Survival was evaluated with the Kaplan-Meier method, and comparisons between groups were made by a log-rank (Mantel-Cox) test. Statistical significance was defined as a *p* value less than 0.05.

## 3. Results

### 3.1. Murine Baseline Characteristics and CA/CPR-Related Variables

**Figure 1:**
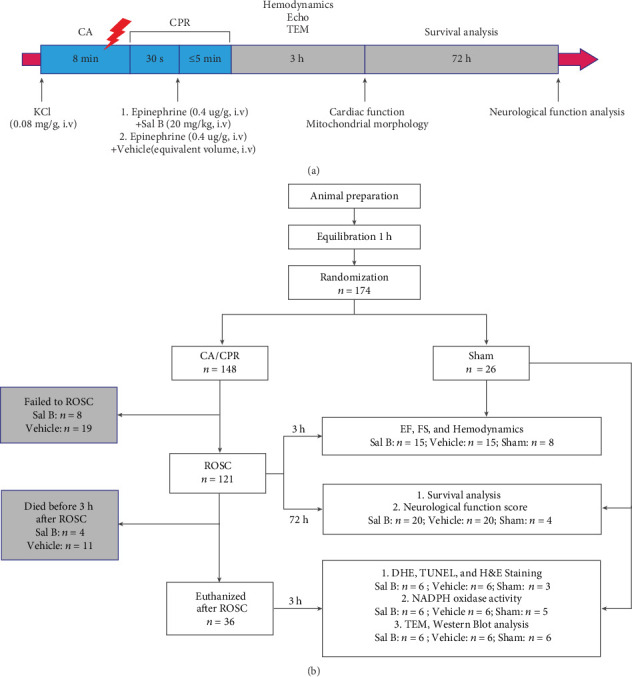
(a) Schematic diagram indicating the time course of the experimental procedure. (b) Flow chart of the experimental groups. KCl: potassium chloride; CA: cardiac arrest; CPR: cardiopulmonary resuscitation; Echo: echocardiography; TEM: transmission electron microscopy; Vehicle: mice subjected to CA/CPR treated with normal saline; Sal B: salvianolic acid B; *n*: number of mice; ROSC: return of spontaneous circulation; h: hours; min: minutes; EF: ejection fraction; FS: fractional shortening; DHE: dihydroethidium; H&E: hematoxylin-eosin; TUNEL: terminal dUTP nick-end labeling; NADPH: nicotinamide adenine dinucleotide phosphate.

An illustration of the experimental procedure used is provided in Figure 1(a). Mice were acclimated to their surroundings for 1 hour before experimentation and then were randomized to the vehicle or Sal B groups by using a random number table. The present study included three parts. In the first part, animals that successfully resuscitated were followed for 72 hours, and the survival rate and neurological outcomes were evaluated. In the second part, the cardioprotective effect of Sal B in the acute phase (three hours) was investigated by echocardiography and hemodynamics. In the third part, in order to clarify the potential mechanism underlying the Sal B-mediated improvement of cardiac function, the relationship between oxidative stress and the Nrf2 signaling pathway was determined.

A total of 148 mice underwent CA/CPR following a standardized protocol, and 26 mice received a sham operation (no CA). Fifteen mice that resuscitated from CA but failed to survive for three hours were excluded from this study, with 11 and four mice in the vehicle and Sal B-treated groups, respectively. The remaining 106 CA/CPR mice were then subjected to either three hours (*n* = 66) or 72 hours (*n* = 40) of investigation, depending on the experimental design (Figure 1(b)). No significant differences were detected in the physiologic variables studied at baseline, and the hemodynamic measurements of the two groups during the pre-CA period were similar. For resuscitation-related variables, there were no differences in resuscitation quality as evaluated by chest compression rate and mean aortic pressure in both CA/CPR groups. Notably, the ROSC rate was comparable between the vehicle and Sal B groups (73% vs. 86%, *p* = 0.648), whereas the time required for ROSC in animals that successfully resuscitated was significantly improved in the Sal B-treated group (*p* = 0.013, Table 1).

### 3.2. Sal B Treatment Attenuated Myocardial Dysfunction and Improved Survival Rate after CA/CPR

**Figure 2:**
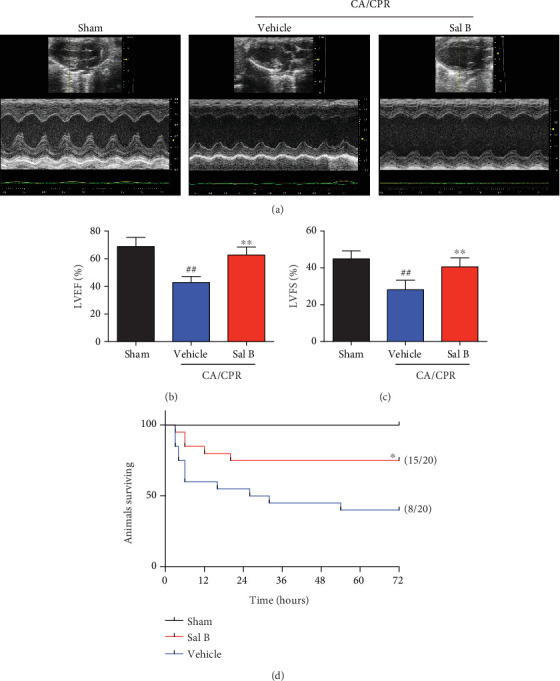
Salvianolic acid B treatment improved cardiac dysfunction and increased survival following cardiac arrest and cardiopulmonary resuscitation (CA/CPR). (a) Cardiac function was evaluated by echocardiography 3 hours following resuscitation. (b, c) Quantitative analysis of LVEF and LVFS in the indicated groups (*n* = 8 per group). Statistical significance was determined using one-way ANOVA followed by post-hoc Bonferroni correction.^##^*p* < 0.01 vs. sham-operated controls; ^∗∗^*p* < 0.01 vs. vehicle group. (d) Kaplan-Meier survival curve of WT and Sal B-treated WT mice after successful CA/CPR within 72 hours (*n* = 20 per group). The number of surviving animals is shown to the right in brackets. ^∗^*p* < 0.05 vs. Vehicle by log-rank test. WT: wild type; LVEF: left ventricular ejection fraction; LVFS: left ventricular fractional shortening.

The effect of Sal B in decreasing ROSC time after CA suggested the involvement of Sal B in the CPR period. To determine whether Sal B exerts cardioprotective effects against CA-induced myocardial injury, vehicle- and Sal B-treated mice were subjected to CA/CPR. Myocardial function, as indicated by LVEF and LVFS, was evaluated by ventricular transthoracic echocardiography in the indicated groups (Figure 2(a)). Eight minutes of CA was associated with remarkable depression of cardiac LVEF and LVFS three hours postresuscitation. However, both LVEF and LVFS were significantly impaired in the vehicle-treated mice when compared to Sal B treatment (42.7 ± 4.3% vs. 62.6 ± 5.7%, *p* < 0.01 and 28.1 ± 5.2% vs. 40.6 ± 4.9%, *p* < 0.01, respectively). Of note, there were no significant differences in LVEF and LVFS between Sal B treatment and sham-operated mice at baseline (Figures 2(b) and 2(c)).

Survival was recorded in the two groups for 72 hours, with a survival rate of 75% (15 of 20) in the Sal B group, which was much higher than that in the vehicle group (40%, 8 of 20; *p* = 0.03). The Kaplan-Meier survival curve also showed a more rapid decline of the survival rate in the vehicle group within the early post-CPR period. The differences in survival rate persisted until 72 hours after CA/CPR (Figure 2(d)). Altogether, these results indicate that Sal B can preserve cardiac function with improved survival after CA/CPR when it is administered during the period of CPR.

### 3.3. Sal B Treatment Improved Cardiovascular Hemodynamics after CA/CPR

**Figure 3:**
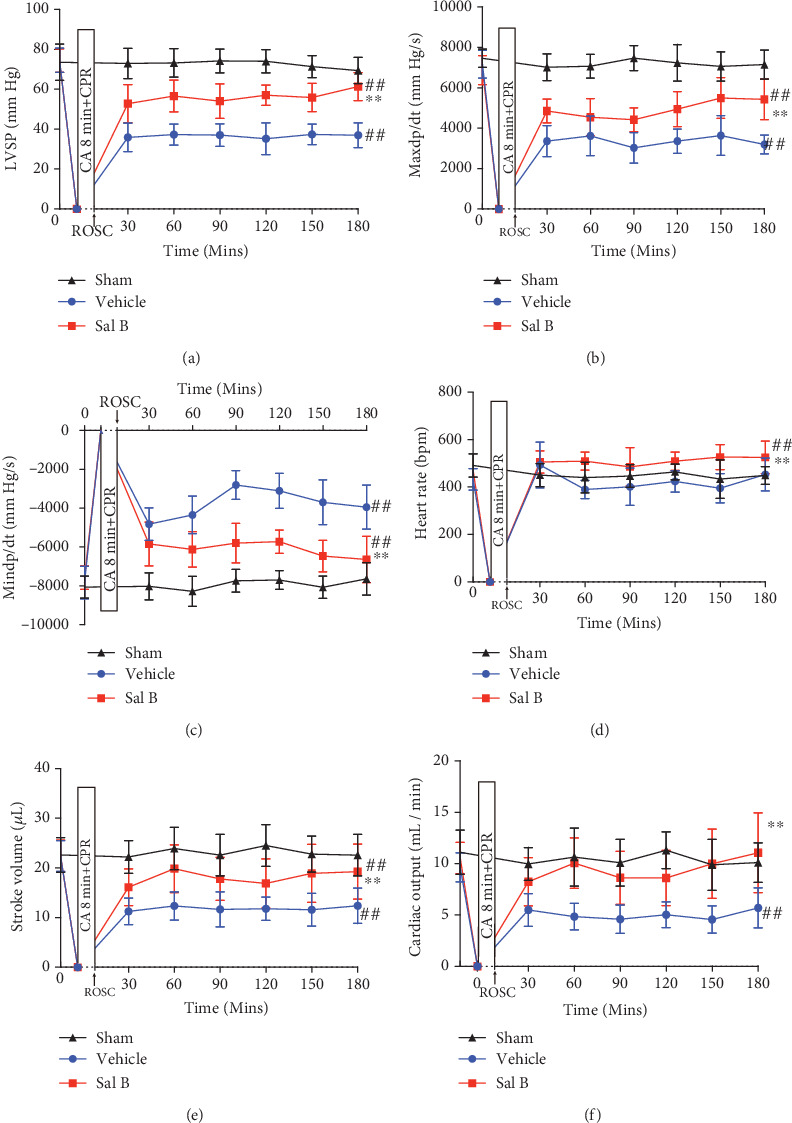
Salvianolic acid B treatment improved cardiovascular hemodynamics following cardiac arrest. Hemodynamic parameters of vehicle and salvianolic acid B-treated groups from baseline to 3 hours after cardiac arrest and cardiopulmonary resuscitation (CA/CPR) were determined by a Millar catheter. (a) Maximum left ventricular systolic pressure (LVSP); (b) dp/dt_max_; (c) dp/dt_min_; (d) heart rate; (e) stroke volume; (f) cardiac output (*n* = 15 per group). ^##^*p* < 0.01 vs. sham-operated controls; ^∗∗^*p* < 0.01 vs. vehicle group. Statistical significance was determined using repeated-measures two-way ANOVA followed by Bonferroni comparison post-hoc test. Mins: minutes; bpm: beats per minute; ROSC: return of spontaneous circulation.

To further confirm Sal B's myocardial effects following CA, we performed dynamic monitoring of myocardial hemodynamics with a Millar catheter. Significant differences in hemodynamic measurements between study groups were not observed before the induction of CA as described in Table 1. The left ventricular systolic pressure (LVSP) depressed following CA/CPR in both groups. However, LVSP was higher in the Sal B-treated group compared to the vehicle group from one to three hours following CPR (*p* < 0.01; Figure 3(a)). Furthermore, the left ventricular systolic function, calculated as dp/dt_max_, and the diastolic function, represented by dp/dt_min_, were remarkably better in the Sal B group compared to the vehicle mice (*p* < 0.01, respectively; Figures 3(b) and 3(c)). Additionally, heart rate, stroke volume, and cardiac output were higher in the Sal B-treated group during hours one to three after CPR (*p* < 0.01, respectively; Figures 3(d)–3(f)). It is worth noting that the cardiac output of Sal B-treated mice within three hours after postresuscitation was preserved similarly to sham-operated mice (*p* = 0.08; Figure 3(f)). Together, these results suggest that Sal B treatment improved cardiac contractility and diastolic function in response to CA/CPR injury.

### 3.4. Sal B Treatment Reduced Myocardial Injuries, Inhibited Myocardial Apoptosis, and Diminished Mitochondrial Damage in Response to CA/CPR

**Figure 4:**
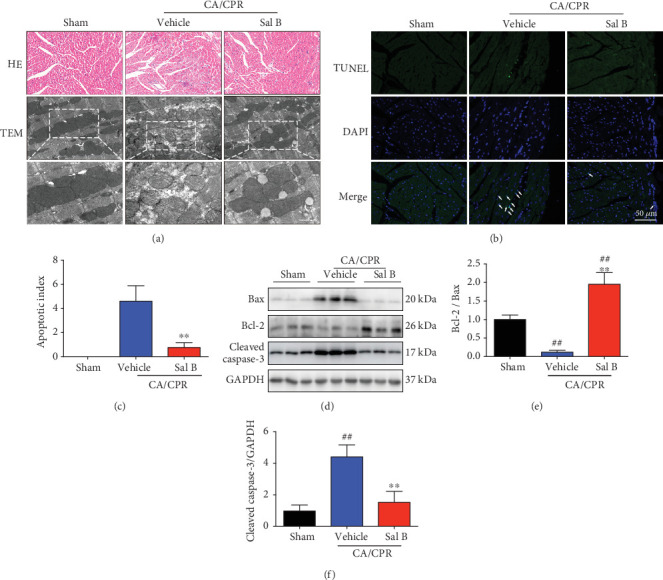
Salvianolic acid B treatment inhibited myocytolysis and myocardial apoptosis, and preserved mitochondrial morphology when challenged with cardiac arrest and cardiopulmonary resuscitation (CA/CPR). (a) H&E staining revealed less myocytolysis and weaving in the myocardium of Sal B-treated mice (top panel, *n* = 6 per group). Transmission electron microscopy was performed to observe the ultrastructure of post-CA myocardial tissues. CA/CPR damaged the myocardial ultrastructure with mitochondrial swelling, outer membrane rupture, and disarrangement of the inner membrane cristae, whereas these changes were reversed by administration of Sal B (bottom panel, *n* = 6 per group). (b) The apoptotic cell death 3 hours post-ROSC was determined using TUNEL. TUNEL (green), apoptotic nuclei; DAPI (blue), and total nuclei. (c) The apoptosis index was quantified and compared between vehicle- and Sal B-treated groups (*n* = 5 − 6 per group). (d) Western blot analysis of Bax, Bcl-2, and cleaved caspase-3 from the indicated groups (*n* = 6 per group). (e) The ratio of Bcl-2/Bax was determined from the Bcl-2 and Bax protein band densities and converted to fold change relative to the sham-operated group. (f) Quantitative value of protein expression levels of cleaved caspase-3. ^##^*p* < 0.01 vs. sham-operated controls; ^∗∗^*p* < 0.01 vs. vehicle group. H&E: hematoxylin-eosin; TEM: transmission electron microscopy; ROSC: return of spontaneous circulation; TUNEL: terminal dUTP nick-end labeling.

Histological studies revealed that there was remarkably less myocytolysis following Sal B administration (Figure 4(a), upper panel). Electron microscopic analysis of the left ventricle in the vehicle group revealed mitochondrial swelling and edema, outer membrane rupture, and loss of inner-membrane cristae with amorphous densities. However, in the Sal B-treated group, the cellular and extracellular architecture of the myocardium was intact, with morphologically normal mitochondria in response to CA/CPR (Figure 4(a), bottom panel). TUNEL assay indicated that cardiomyocyte apoptosis increased in the vehicle group when compared to the sham group, which was significantly suppressed by Sal B administration in the Sal B+CA/CPR group mice (Figure 4(b)). Quantitative results, using the apoptotic index, are shown in Figure 4(c). Lower percentages of apoptotic cells were found in Sal B-treated group (0.8 ± 0.4%) compared to the vehicle mice (4.6 ± 1.2%; *p* < 0.01). Therefore, we investigated the expression of several proapoptotic and antiapoptotic genes implicated in apoptosis pathway regulation, including Bax and Bcl-2 (Figure 4(d)). The upregulation of the antiapoptotic gene (Bcl-2) and downregulation of the proapoptotic gene (Bax) were induced by Sal B treatment, leading to the relative increase of the Bcl-2/Bax ratio compared to the vehicle mice (Figure 4(e)). Because caspase-3 is the critical mediator of apoptosis, the protein levels of cleaved caspase-3, the active form of caspase-3, were detected by Western Blot analysis (Figure 4(d)). The upregulated cleaved caspase-3 levels induced by CA/CPR were suppressed by the treatment of Sal B (Figure 4(f)). These results demonstrated that Sal B treatment exerted a cardioprotective effect against CA/CPR-induced myocardial injuries and apoptosis.

### 3.5. Sal B Treatment Attenuated Oxidative Stress in the Heart following CA/CPR

**Figure 5:**
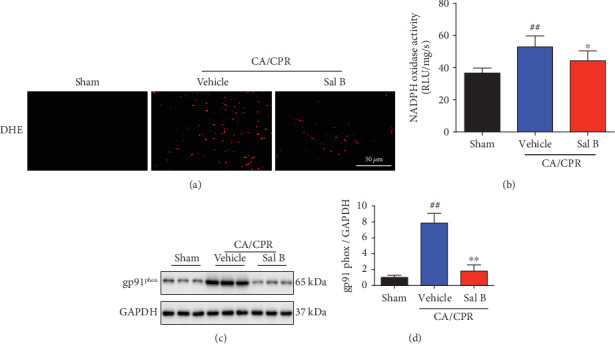
Salvianolic acid B treatment suppressed the superoxide production in the heart following cardiac arrest and cardiopulmonary resuscitation (CA/CPR). (a) ROS steady-state levels in the myocardium were determined by in situ detection of superoxide with dihydroethidium fluorescence among different groups (*n* = 3 − 6 per group). (b) NADPH oxidase activity was assessed using a lucigenin assay 3 hours following CA/CPR (*n* = 5 − 6 per group). (c, d) gp91^phox^ expression was determined using Western blot analysis. GAPDH was used as a loading control and results were converted to the fold change relative to the sham-operated group (*n* = 6 per group). Statistical significance was determined using one-way ANOVA followed by post-hoc Bonferroni correction. ^##^*p* < 0.01 vs. sham-operated controls; ^∗^*p* < 0.05 or ^∗∗^*p* < 0.01 vs. vehicle group. ROS: reactive oxygen species; DHE: dihydroethidium; NADPH: nicotinamide adenine dinucleotide phosphate.

Oxidative damage is a known consequence of CA and is likely a contributing factor to myocardial dysfunction. Mitochondria represent both a major source of ROS production and the primary target of oxidative damage [27]. To determine the mechanisms underlying the protective actions of Sal B, we next investigated the role of Sal B in regulating CA/CPR-induced oxidative stress. Sal B dramatically attenuated oxidative stress, as evidenced by the reduced generation of superoxide (Figure 5(a)) and inhibited NADPH oxidase activity (Figure 5(b)). Moreover, Sal B downregulated the expression of gp91^phox^, the major component of NADPH oxidase responsible for the generation of superoxide anions, in the heart following CA/CPR (Figures 5(c) and 5(d)). Taken together, these results suggest that the attenuation of myocardial injury by Sal B is due to the protection of mitochondria against oxidative stress.

### 3.6. Sal B Treatment Reduced ROS Production via the Activation of Nrf2 Signaling Pathway

**Figure 6:**
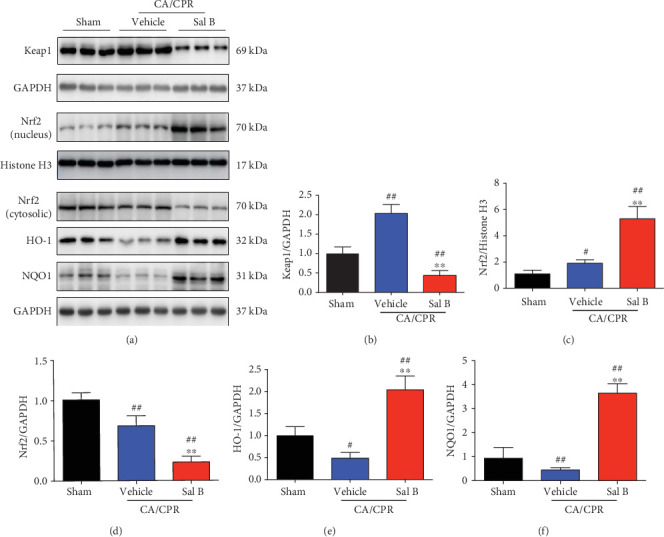
Salvianolic acid B treatment activated the Nrf2 antioxidative pathway after cardiac arrest and cardiopulmonary resuscitation (CA/CPR). (a) Expression of Keap1, nuclear and cytosolic Nrf2, HO-1, and NQO1 in the myocardium 3 hours following CA/CPR with or without Sal B treatment. (b–f) Western blot analysis and quantification of Keap1, nuclear and cytosolic Nrf2, HO-1, and NQO1 in the indicated groups. Histone H3 and GAPDH served as loading controls and results were converted to fold change relative to the sham-operated group (*n* = 6 per group). ^#^*p* < 0.05 or ^##^*p* < 0.01 vs. sham-operated controls; ^∗∗^*p* < 0.01 vs. vehicle group.

Next, we examined the specific mechanism by which Sal B regulates oxidative stress. NF-E2-related factor 2 (Nrf2), the main antioxidant transcriptional regulator that binds to antioxidant response elements, is responsible for the elimination of oxidative toxicants [28]. Previous studies reported that Sal B involved in the regulation of Nrf2 signaling pathway to counteract oxidative damage [20–22]. To investigate whether Sal B attenuated oxidative stress in the heart after CA/CPR via the Nrf2 signaling pathway, we performed Western blots to analyze the expressions of Nrf2-pathway-related proteins (Figure 6(a)). Kelch ECH associating protein 1 (Keap1), a cytosolic repressor of the Nrf2 pathway, was significantly decreased in Sal B-treated group when compared to the vehicle group (Figure 6(b)). We then evaluated the expression of Nrf2 in the nucleus and cytosol. Sal B treatment significantly enhanced the nuclear Nrf2 level and its level in the cytoplasm was downregulated accordingly (Figure 6(c) and 6(d)). Meanwhile, Western blot demonstrated that CA/CPR insult considerably reduced the Nrf2 downstream antioxidant genes HO-1 and NQO1, all of which were restored by Sal B treatment (Figures 6(e) and 6(f)). Collectively, these results suggest the inhibition of oxidative stress by Sal B following CA/CPR is probably attributive to the activation of the Nrf2 antioxidative pathway, which facilitated cardiomyocyte survival and improved cardiac function.

### 3.7. Gene Silencing of Nrf2 Abrogated the Protective Effect of Sal B on ROS Production and Apoptosis In Vitro

**Figure 7:**
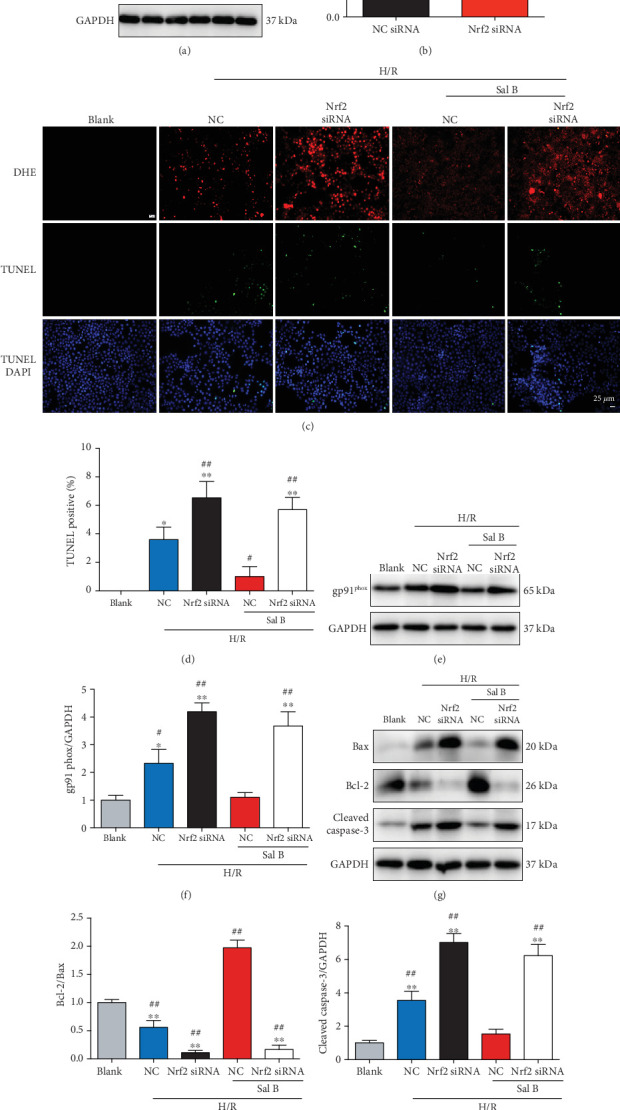
Knockdown of Nrf2 abolished the antioxidative and antiapoptotic effects of Sal B treatment in vitro. (a, b) Protein expression of Nrf2 was assessed using immunoblot 72 hours after Nrf2 siRNA or NC siRNA transfection in H9c2 cardiomyocytes. ^##^*p* < 0.01 vs. NC siRNA. (c) Representative fluorescent images of DHE and TUNEL staining in the presence of Nrf2 siRNA or NC siRNA with or without Sal B treatment (10 *μ*M) in H9c2 cardiomyocytes after H/R insult. (d) Quantification of the number of TUNEL-positive cells in the indicated groups. (e, f) gp91^phox^ expression was determined using Western blot analysis. (g) Western blot analysis of Bax, Bcl-2, and cleaved caspase-3 from the indicated groups. (h) The ratio of Bcl-2/Bax was determined from the Bcl-2 and Bax protein band densities and converted to fold change relative to Blank. (i) Quantitative value of protein expression levels of cleaved caspase-3. GAPDH served as the internal control and results were converted to fold change relative to Blank. ^#^*p* < 0.05 or ^##^*p* < 0.01 vs. Blank; ^∗^*p* < 0.05 or ^∗∗^*p* < 0.01 vs. NC+Sal B. NC: negative control siRNA; H/R: hypoxia/reoxygenation.

To further confirm the involvement of the Nrf2 pathway in the protective role of Sal B treatment, H9c2 cardiomyocytes were transfected with Nrf2 siRNA or NC siRNA. Western blot showed that Nrf2 siRNA transfection led to a significant reduction in Nrf2 protein levels (Figures 7(a) and 7(b)). A model of H/R injury in H9c2 cardiomyocytes was established in our study. DHE staining revealed that Sal B treatment reduced the superoxide production, but the antioxidative effects were attenuated by Nrf2 knockdown (Figure 7(c)). The Western blot analysis also demonstrated that Nrf2 knockdown blocked the suppressive effect of Sal B on the gp91^phox^ upregulation in response to H/R insult (Figures 7(e) and 7(f)). Furthermore, Nrf2 silencing diminished the inhibitory effects of Sal B treatment on H/R-induced apoptosis, with a higher percentage of apoptotic cells (Figures 7(c) and 7(d)), a decrease in the Bcl-2/Bax ratio (Figures 7(g) and 7(h)), and the activation of cleaved caspase-3 (Figures 7(g) and 7(i)) when compared to the Sal B+NC group. These results provided evidence in vitro that Nrf2 activation is essential for Sal B to combat oxidative stress and facilitate cell survival under hypoxic conditions. Taken together, these results confirmed the involvement of the Nrf2 pathway in the protective role of Sal B on ROS production and apoptosis.

### 3.8. Sal B Treatment Improved Neurological Outcomes after CA/CPR

**Figure 8:**
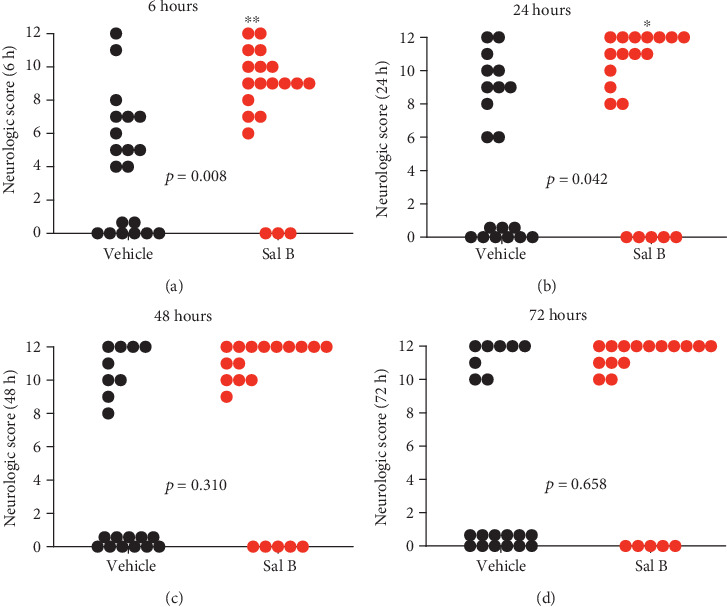
The neurological function score was assessed in the surviving mice within 72 hours at indicated time points after cardiac arrest and cardiopulmonary resuscitation (CA/CPR). (a) Six hours after ROSC. (b) 24 hours after ROSC. (c) 48 hours after ROSC. (d) 72 hours after ROSC (*n* = 20 per group). Each circle indicates an individual animal scored after resuscitation. Dead mice, indicated by score 0, were excluded from the statistical analysis. ^∗^*p* < 0.05 or ^∗∗^*p* < 0.01 between the two groups (unpaired t-test). ROSC: return of spontaneous circulation.

Neurological damage post-CA is a major contributor to post-CA morbidity and mortality [29, 30]. The recovery of neurological function in the surviving mice, determined by the neurological functioning score system, greatly improved in the Sal B-treated group at six and 24 hours following CA/CPR (*p* = 0.008 and *p* = 0.042, Figures 8(a) and 8(b), respectively). However, no significant differences were detected between the two groups in the neurological scores at 48 and 72 hours after CA/CPR (Figures 8(c) and 8(d)), although the vehicle group showed a higher overall mortality rate. These data demonstrate that Sal B treatment not only improved survival following CA/CPR, but was also associated with favorable neurological outcomes.

## 4. Discussion

In the present study, we found that the administration of Sal B very early in CPR markedly improved myocardial and neurological function during the postresuscitation period. The suppression of oxidative stress by Sal B had cardioprotective effects and reduced myocardial injury, and may contribute to improving post-CA survival outcomes.

CA-induced myocardial dysfunction and hemodynamic instability are responsible for the poor survival rate following resuscitation [31, 32]. Some studies have indicated that myocardial dysfunction is reversible after successful resuscitation, which differs from the decompensation of acute cardiac failure [33]. However, insufficient cardiac output and poor peripheral circulation in the early postresuscitation period may deteriorate global I/R injuries [34]. Increased support of the circulatory system during CPR and the early postresuscitation period can stabilize hemodynamics and improve survival outcomes [35]. Accumulating evidence has demonstrated that Sal B can protect against acute or chronic decompensated heart failure. However, the effect of Sal B on post-CA myocardial dysfunction has not been reported previously. Our study suggests that Sal B treatment exerted positive inotropic and lusitropic effects in the murine CA model. Sal B was able to improve and stabilize circulatory failure induced by CA, resulting in better survival outcomes in the postresuscitation period. Microscopically, we found that Sal B treatment attenuated myocytolysis and inhibited cardiomyocyte apoptosis with preserved mitochondrial morphology in this model. It is noteworthy that whether apoptosis is one of the main mechanisms of postresuscitation myocardial dysfunction remains controversial [36–39]. The experimental animals and the inducers of CA could be driving these differences.

The vicious cycle of oxidative damage is a known contributor to myocardial apoptosis that impairs cardiac function following I/R injury [40]. CA is defined as a global ischemic event followed by whole-body reperfusion when ROSC is successfully achieved. Oxidative stress triggered by a whole-body I/R injury associated with CA/CPR exacerbates myocardial injury, as the restoration of oxygen supply to circulation leads to the formation of ROS [41]. The consequent ROS increase activates the mitochondria-mediated apoptotic cell death pathway, contributing to impaired cardiac performance [42]. Previous studies have suggested that mitigating ROS production by administration of antioxidants such as vitamin C following ROSC was able to reduce myocardial injury, resulting in improved postresuscitation myocardial function [43, 44]. Because oxidative stress is causatively related to mitochondrial dysfunction, we determined the role of Sal B in the regulation of oxidative stress. The involvement of Sal B in regulating oxidative stress has been reported in several cell types; interestingly, Sal B had antioxidant effects in several cell types, but oxidative effects in others. For example, Sal B inhibited oxidative stress in endothelial cells, smooth muscle cells, and Schwann cells [45–47], but facilitated oxidative stress in some tumor cells, such as glioma, osteosarcoma, and colorectal carcinomas [48–50]. Thus, the role of Sal B in inhibiting or promoting oxidative stress may be dependent on cell and tissue type. There is growing evidence that Sal B exerts cardioprotective effects through inhibition of oxidative stress [51]. As an antioxidant, Sal B is able to efficiently eliminate free radicals and superoxide anions with higher scavenging activity than vitamin C [52], which prompted us to determine its antioxidant capacity in CA/CPR. In our study, we observed that CA/CPR markedly upregulated the expression of NADPH oxidase and exacerbated superoxide generation in the heart of WT mice but not in WT mice supplemented with Sal B. These observations suggest that Sal B treatment prevents oxidative stress with preserved mitochondrial function, which indicates a correlation between oxidative stress and myocardial dysfunction after CA/CPR.

Nrf2, as a major antioxidant transcription factor, could bind to antioxidant response elements of over 600 antioxidant enzymes to protect against oxidative stress. Nrf2 is regulated by a complicated system of protein turnover, among which Keap1 is a main regulator. Keap1 could bind with Nrf2 and promote its degradation rapidly in a proteasome-dependent manner in the cytoplasm. Under oxidative stress conditions, Keap1 is inactivated, causing the release of Nrf2 from Keap1 [53]. The activation of the Nrf2 signaling pathway in response to oxidative stress is central in myocardial cardioprotection against CA/CPR-induced injury [54]. However, the role of Nrf2 in Sal B cardioprotection in ischemic heart disease has not been defined. Our study demonstrated that the Sal B administration inhibited Keap1 expression and promoted Nrf2 nuclear translocation, which induced its downstream factors HO-1 and NQO1 to reverse oxidative damage. Furthermore, loss of function study in vitro confirmed that the Nrf2 signaling pathway is essential to mediate the protection of Sal B treatment from ROS-induced cardiomyocyte apoptosis. These data are in line with the findings of the previous reports illustrating the activating effects of Sal B on the Nrf2 signaling pathway and its target genes [20–22].

The severity of post-CA myocardial dysfunction is associated with poor neurological outcomes, and a majority of mortality and morbidity after CA/CPR is caused by global ischemic brain injury [29]. The brain is highly sensitive to ischemic conditions. Even a brief period of loss of blood flow during CA may directly result in neuronal damage in vulnerable regions of the brain. Secondary neurological injuries that result from derangements of recirculation have also been described after successful CPR [55]. The ischemic brain injury cascade contributing to the activation of cell death signaling pathways is mediated by multiple processes, including rapidly depleted ATP, increased excitatory amino acids, disrupted calcium homeostasis, ROS generation, and pathological protease cascade [56]. Previous studies have demonstrated a beneficial effect of Sal B on neuronal activity in cerebral ischemia injury, Parkinson's disease, and Alzheimer's disease [20, 57]. Clinical trials have also reported that Danshen tended to improve the short-term effect of ischemic stroke, although this effect was found to be lesser or not significant in several trials [58, 59]. Future prospective clinical trials with larger sample sizes and longer study durations should be conducted to further validate the impact of Danshen or its independent ingredients on ischemic stroke. To our knowledge, no study has yet investigated the potential effect of Sal B, the key component of Danshen, on neural function induced by whole-body ischemia. In the present study, we found that severe neurological disorders were detected in mice subjected to CA/CPR compared to sham-operated animals. However, it is worth noting that an improvement of neurological performance after CA/CPR by a single dose of Sal B was observed within 72 hours post-ROSC in addition to the abovementioned positive effects on myocardial hemodynamics in our model. These neurological benefits of Sal B treatment after CA/CPR could be due to the rapid, improved recovery of stable hemodynamics and cerebral blood perfusion. However, it cannot rule out the possibility that Sal B exerts direct neuroprotective effects, given its roles in other neurological pathologies.

Our study has several limitations. First, mice were used as experimental subjects, and future studies should utilize larger animals such as pigs or dogs to determine the protective effects of Sal B during CA/CPR. Second, CA was induced by a bolus administration of KCl and performed on healthy animals with no underlying heart failure or coronary disease, which may have reduced its clinical relevance. However, we believe that this CA model provides a valuable platform for investigating the specific effects of Sal B treatment on the post-CA syndrome. More studies are therefore needed to further clarify the effects of Sal B on diseased animal models. Third, although we focused here on the cardioprotective effects of Sal B, it may also exert effects on other organs, and these possible beneficial effects remain to be determined. Fourth, clinical trials have indicated that therapeutic hypothermia confers improved neurologic outcomes and survival benefits when it is applied minutes to hours after successful CPR for CA [60, 61]. However, in order to initially elucidate the net effects of Sal B administration on post-CPR myocardial dysfunction, hypothermia treatment was not utilized in our study.

Based on the ability of Sal B treatment to improve outcomes after CA/CPR, it may be a practical therapeutic agent that can be initiated after patients are transferred to a hospital. We anticipate that the rapid translation of animal model findings of the Chinese traditional medicine Sal B will provide new strategies to better treat patients suffering from the post-CA syndrome.

## 5. Conclusions

The present study revealed robust protective effects of Sal B treatment on CA/CPR, which could have therapeutic implications given the paucity of powerful pharmacological strategies.

## Figures and Tables

**Table 1:** Baseline and CA/CPR-related characteristics of mice.

Variables	Vehicle *n* = 15	Sal B *n* = 15	*p* value
Baseline			
Body weight (g)	23.0 ± 1.3	22.5 ± 1.2	0.257
Body temperature (°C)	36.8 ± 0.4	36.9 ± 0.3	0.880
LVP_max_ (mmHg)	73 ± 7	73 ± 9	0.943
dp/dt_max_ (mmHg/s)	6347 ± 906	5901 ± 860	0.177
dp/dt_min_ (mmHg/s)	−6403 ± 875	−6185 ± 718	0.463
Heart rate (bpm)	452 ± 41	455 ± 26	0.760
Cardiac output (mL/min)	9.6 ± 1.4	10.5 ± 1.5	0.101
Resuscitation			
Intubation time (s)	32 ± 7	33 ± 7	0.837
Chest compression rate (beats/min)	374 ± 10	376 ± 15	0.508
Mean arterial pressure (mmHg)	54 ± 7	57 ± 6	0.649
Total dose of epinephrine (*μ*g)	9.2 ± 0.5	9.0 ± 0.4	0.257
Time to ROSC (s)	251 ± 22	227 ± 26	0.013^a^
ROSC success (*n* (%))	11 (73%)	13 (86%)	0.648

Values are means ± SD; CA: cardiac arrest; CPR: cardiopulmonary resuscitation; Vehicle: mice subjected to CA/CPR treated with normal saline; Sal B: salvianolic acid B; *n*: number of mice; LVP_max_: maximum left ventricular pressure; dp/dt_max_: maximum rate of developed left ventricular pressure; dp/dt_min_: minimum rate of developed left ventricular pressure; bpm: beats per minute; ROSC: return of spontaneous circulation. ^a^*p* < 0.05 vs. Vehicle.

## Data Availability

The data used to support the findings of this study are included within the article.

## References

[1] Benjamin E. J., Muntner P., Alonso A. (2019). Heart disease and stroke Statistics-2019 update: a report from the American Heart Association. *Circulation*.

[2] Fang X. S., Hao J. F., Zhou H. Y., Zhu L. X., Wang J. H., Song F. Q. (2010). Pharmacological studies on the sedative-hypnotic effect of *Semen Ziziphi spinosae* (Suanzaoren) and *Radix et Rhizoma Salviae miltiorrhizae* (Danshen) extracts and the synergistic effect of their combinations. *Phytomedicine*.

[3] Yan W. J. P. P., Remedies C. (2011). Effect of compound danshen dripping pill on primary dysmenorrhea. *Practical Pharmacy and Clinical Remedies*.

[4] Xu G., Zhao W., Zhou Z., Zhang R., Zhu W., Liu X. (2009). Danshen extracts decrease blood C reactive protein and prevent ischemic stroke recurrence: a controlled pilot study. *Phytotherapy Research*.

[5] Fan Y., Yuan Y., Feng Z. (2009). Effect of entecavir combined with compound danshen dripping pill on hepatic fibrosis induced by chronic hepatitis B. *Journal of Hainan Medical College*.

[6] Lam F. F. Y., Yeung J. H. K., Chan K. M., Or P. M. Y. (2008). Dihydrotanshinone, a lipophilic component of _Salvia miltiorrhiza_ (danshen), relaxes rat coronary artery by inhibition of calcium channels. *Journal of Ethnopharmacology*.

[7] Kim D. D., Sánchez F. A., Durán R. G., Kanetaka T., Durán W. N. (2007). Endothelial nitric oxide synthase is a molecular vascular target for the Chinese herb Danshen in hypertension. *American Journal of Physiology. Heart and Circulatory Physiology*.

[8] Guo J., Zhang L., Jiang Y. (2005). Effects of compound Dan-shen root dropping pill on hemorheology in high-fat diet induced hyperlipidemia in dogs. *Clinical Hemorheology and Microcirculation*.

[9] Qian Q., Qian S., Fan P., Huo D., Wang S. (2012). Effect of Salvia miltiorrhiza hydrophilic extract on antioxidant enzymes in diabetic patients with chronic heart disease: a randomized controlled trial. *Phytotherapy Research*.

[10] Pan C. S., Liu Y. H., Liu Y. Y. (2015). Salvianolic acid B ameliorates lipopolysaccharide-induced albumin leakage from rat mesenteric venules through Src-regulated transcelluar pathway and paracellular pathway. *PLoS One*.

[11] Yue J., Li B., Jing Q., Guan Q. (2015). Salvianolic acid B accelerated ABCA1-dependent cholesterol efflux by targeting PPAR-*γ* and LXR*α*. *Biochemical and Biophysical Research Communications*.

[12] Deng Y., Yang M., Xu F. (2015). Combined Salvianolic acid B and ginsenoside Rg1 exerts cardioprotection against ischemia/reperfusion injury in rats. *PLoS One*.

[13] Ling C., Liang J., Zhang C. (2018). Synergistic effects of salvianolic acid B and puerarin on cerebral ischemia reperfusion injury. *Molecules*.

[14] Kong R., Gao Y., Sun B. (2009). The strategy of combined ischemia preconditioning and salvianolic acid-B pretreatment to prevent hepatic ischemia-reperfusion injury in rats. *Digestive Diseases and Sciences*.

[15] Ma Z. G., Xia H. Q., Cui S. L., Yu J. (2017). Attenuation of renal ischemic reperfusion injury by salvianolic acid B via suppressing oxidative stress and inflammation through PI3K/Akt signaling pathway. *Brazilian Journal of Medical and Biological Research*.

[16] Suzuki T., Yamamoto M. (2015). Molecular basis of the Keap1-Nrf2 system. *Free Radical Biology and Medicine*.

[17] Sun Z., Zhang S., Chan J. Y., Zhang D. D. (2007). Keap1 controls postinduction repression of the Nrf2-mediated antioxidant response by escorting nuclear export of Nrf2. *Molecular and Cellular Biology*.

[18] Sykiotis G. P., Bohmann D. (2010). Stress-activated cap'n'collar transcription factors in aging and human disease. *Science Signaling*.

[19] Chen Q. M., Maltagliati A. J. (2018). Nrf2 at the heart of oxidative stress and cardiac protection. *Physiological Genomics*.

[20] Zhang X., Wu Q., Lu Y. (2018). Cerebroprotection by salvianolic acid B after experimental subarachnoid hemorrhage occurs via Nrf2- and SIRT1-dependent pathways. *Free Radical Biology and Medicine*.

[21] Liu X., Xavier C., Jann J., Wu H. (2016). Salvianolic acid B (Sal B) protects retinal pigment epithelial cells from oxidative stress-induced cell death by activating glutaredoxin 1 (Grx1). *International Journal of Molecular Sciences*.

[22] Tongqiang L., Shaopeng L., Xiaofang Y. (2016). Salvianolic acid B prevents iodinated contrast media-induced acute renal injury in rats via the PI3K/Akt/Nrf2 pathway. *Oxidative Medicine and Cellular Longevity*.

[23] Abella B. S., Zhao D., Alvarado J., Hamann K., vanden Hoek T. L., Becker L. B. (2004). Intra-arrest cooling improves outcomes in a murine cardiac arrest model. *Circulation*.

[24] Deng G., Orfila J. E., Dietz R. M. (2017). Autonomous CaMKII activity as a drug target for histological and functional neuroprotection after resuscitation from cardiac arrest. *Cell Reports*.

[25] Xue L., Wu Z., Ji X. P., Gao X. Q., Guo Y. H. (2014). Effect and mechanism of salvianolic acid B on the myocardial ischemia- reperfusion injury in rats. *Asian Pacific Journal of Tropical Medicine*.

[26] Ji Q., Zhao Y., Yuan A., Pu J., He B. (2017). Deficiency of liver-X-receptor-*α* reduces glucose uptake and worsens post- myocardial infarction remodeling. *Biochemical and Biophysical Research Communications*.

[27] Ren Y., Li Y., Yan J. (2017). Adiponectin modulates oxidative stress-induced mitophagy and protects C2C12 myoblasts against apoptosis. *Scientific Reports*.

[28] Tonelli C., Chio I. I. C., Tuveson D. A. (2018). Transcriptional regulation by Nrf2. *Antioxidants & Redox Signaling*.

[29] Uray T., Lamade A., Elmer J. (2018). Phenotyping cardiac arrest: bench and bedside characterization of brain and heart injury based on etiology. *Critical Care Medicine*.

[30] Zhang J., Wang Y., Ju M. (2020). Neuroprotective effect of the inhibitor salubrinal after cardiac arrest in a rodent model. *Oxidative Medicine and Cellular Longevity*.

[31] Laurent I., Monchi M., Chiche J. D. (2002). Reversible myocardial dysfunction in survivors of out-of-hospital cardiac arrest. *Journal of the American College of Cardiology*.

[32] Yang J., Xiao Y., Quan E. Y. (2018). Effects of polyethylene glycol-20k on postresuscitation myocardial and cerebral function in a rat model of cardiopulmonary resuscitation. *Critical Care Medicine*.

[33] Ruiz-Bailén M., de Hoyos E. A., Ruiz-Navarro S. (2005). Reversible myocardial dysfunction after cardiopulmonary resuscitation. *Resuscitation*.

[34] Kern K. B., Hilwig R. W., Rhee K. H., Berg R. A. (1996). Myocardial dysfunction after resuscitation from cardiac arrest: an example of global myocardial stunning. *Journal of the American College of Cardiology*.

[35] Morimura N., Sakamoto T., Nagao K. (2011). Extracorporeal cardiopulmonary resuscitation for out-of-hospital cardiac arrest: a review of the Japanese literature. *Resuscitation*.

[36] Gu W., Li C. S., Yin W. P., Guo Z. J., Hou X. M., Zhang D. (2012). Apoptosis is involved in the mechanism of postresuscitation myocardial dysfunction in a porcine model of cardiac arrest. *The American Journal of Emergency Medicine*.

[37] Song F., Shan Y., Cappello F. (2010). Apoptosis is not involved in the mechanism of myocardial dysfunction after resuscitation in a rat model of cardiac arrest and cardiopulmonary resuscitation. *Critical Care Medicine*.

[38] Gu W., Li C., Yin W., Guo Z., Hou X., Zhang D. (2012). Shen-fu injection reduces postresuscitation myocardial dysfunction in a porcine model of cardiac arrest by modulating apoptosis. *Shock*.

[39] Huang C. H., Wang C. H., Tsai M. S. (2016). Urocortin treatment improves acute hemodynamic instability and reduces myocardial damage in post-cardiac arrest myocardial dysfunction. *PLoS One*.

[40] Bolli R., Marban E. (1999). Molecular and cellular mechanisms of myocardial stunning. *Physiological Reviews*.

[41] Yellon D. M., Hausenloy D. J. (2007). Myocardial reperfusion injury. *The New England Journal of Medicine*.

[42] Yao T., Ying X., Zhao Y. (2015). Vitamin D receptor activation protects against myocardial reperfusion injury through inhibition of apoptosis and modulation of autophagy. *Antioxidants & Redox Signaling*.

[43] Tsai M. S., Huang C. H., Tsai C. Y. (2011). Ascorbic acid mitigates the myocardial injury after cardiac arrest and electrical shock. *Intensive Care Medicine*.

[44] Tsai M. S., Huang C. H., Tsai C. Y. (2014). Combination of intravenous ascorbic acid administration and hypothermia after resuscitation improves myocardial function and survival in a ventricular fibrillation cardiac arrest model in the rat. *Academic Emergency Medicine*.

[45] Liu Q., Shi X., Tang L. (2018). Salvianolic acid B attenuates experimental pulmonary inflammation by protecting endothelial cells against oxidative stress injury. *European Journal of Pharmacology*.

[46] Sun L.-Q., Zhao J., Zhang T.-T. (2012). Protective effects of salvianolic acid B on Schwann cells apoptosis induced by high glucose. *Neurochemical Research*.

[47] Zhang H. S., Wang S. Q. (2006). Salvianolic acid B from _Salvia miltiorrhiza_ inhibits tumor necrosis factor- *α* (TNF-*α*)-induced MMP-2 upregulation in human aortic smooth muscle cells via suppression of NAD(P)H oxidase-derived reactive oxygen species. *Journal of Molecular and Cellular Cardiology*.

[48] Guo P., Wang S., Liang W. (2017). Salvianolic acid B reverses multidrug resistance in HCT-8/VCR human colorectal cancer cells by increasing ROS levels. *Molecular Medicine Reports*.

[49] Wang Z.-S., Luo P., Dai S.-H., Liu Z.-B., Zheng X.-R., Chen T. (2013). Salvianolic acid B induces apoptosis in human glioma U87 cells through p38-mediated ROS generation. *Cellular and Molecular Neurobiology*.

[50] Zeng Z., Zhang H., Wang X. (2018). Salvianolic acid B suppresses cell proliferation and induces apoptosis in osteosarcoma through p38-mediated reactive oxygen species generation. *Oncology Letters*.

[51] Tang Y., Jacobi A., Vater C., Zou X., Stiehler M. (2014). Salvianolic acid B protects human endothelial progenitor cells against oxidative stress-mediated dysfunction by modulating Akt/mTOR/4EBP1, p38 MAPK/ATF2, and ERK1/2 signaling pathways. *Biochemical Pharmacology*.

[52] Zhao G. R., Zhang H. M., Ye T. X. (2008). Characterization of the radical scavenging and antioxidant activities of danshensu and salvianolic acid B. *Food and Chemical Toxicology*.

[53] Zheng D., Liu Z., Zhou Y. (2020). Urolithin B, a gut microbiota metabolite, protects against myocardial ischemia/reperfusion injury *via* p62/Keap1/Nrf2 signaling pathway. *Pharmacological Research*.

[54] Xiao X., Hou H., Lin V. (2017). Probucol protects rats from cardiac dysfunction induced by oxidative stress following cardiopulmonary resuscitation. *Oxidative Medicine and Cellular Longevity*.

[55] Tasker R. C., Menon D. K. (2016). Critical care and the brain. *JAMA*.

[56] Reis C., Akyol O., Araujo C. (2017). Pathophysiology and the monitoring methods for cardiac arrest associated brain injury. *International Journal of Molecular Sciences*.

[57] Zhao R., Liu X., Zhang L., Yang H., Zhang Q. (2019). Current progress of research on neurodegenerative diseases of salvianolic acid B. *Oxidative Medicine and Cellular Longevity\*.

[58] Wu B., Liu M., Zhang S. (2007). Dan Shen agents for acute ischaemic stroke. *Cochrane Database of Systematic Reviews*.

[59] Sze F. K., Yeung F. F., Wong E., Lau J. (2005). Does Danshen improve disability after acute ischaemic stroke?. *Acta Neurologica Scandinavica*.

[60] Nordberg P., Taccone F. S., Truhlar A. (2019). Effect of trans-nasal evaporative intra-arrest cooling on functional neurologic outcome in out-of-hospital cardiac arrest: the PRINCESS randomized clinical trial. *JAMA*.

[61] Hackenhaar F. S., Medeiros T. M., Heemann F. M. (2017). Therapeutic hypothermia reduces oxidative damage and alters antioxidant defenses after cardiac arrest. *Oxidative Medicine and Cellular Longevity*.

